# An Essay on Warm, Cold, and Vapour Bathing; with Practical Observations on Sea Bathing, Diseases of the Skin, Bilious, Liver Complaints, and Dropsy

**Published:** 1819-07-01

**Authors:** 


*An Essay on Warm, Cold, and Vapour Bathing; with Practical Observations on Sea Bathing, Diseases of the Skin, Bilious, Liver Complaints, and Dropsy.* By Sir ARTHUR CLARKE, M.D. &c. 4th Edition, small Octavo, pp. 177. London, 1819.

" THOUGH he is himself the proprietor of one of the largest establishments of baths in the United Kingdoms, he trusts it will be found he has discussed the subject with impartiality." *Preface.*

This little work is evidently designed for the general reader, or invalid, rather than for the profession; and therefore it does not fairly come under the laws of medical criticism. Considering that Sir Arthur must have some little partiality for the measures pursued in his extensive establishment, we are ready to acknowledge that there is none of that *ultra-encomium* in his publication, which too often engenders scepticism towards the statements of *ex parte* authors. Sir Arthur has culled freely from the works of others, and *sometimes* forgets the good old maxim—"*Suum cuique.*"

